# Emotion research on education public opinion based on text analysis and deep learning

**DOI:** 10.3389/fpsyg.2022.992419

**Published:** 2022-10-26

**Authors:** Shulin Niu

**Affiliations:** School of Education Science, Zhoukou Normal University, Zhoukou, China

**Keywords:** Education Data Mining, education public opinion, cognitive psychology, emotion classification, EMO-CBOW

## Abstract

Education public opinion information management is an important research focus in the field of Education Data Mining (EDM). In this paper, we classify the education data information based on the traditional Flat-OCC model. From the cognitive psychology perspective, we identify up to 12 kinds of emotions, including sadness and happiness. In addition, the EMO-CBOW model is also proposed in this paper to further identify emotion by using various emoticons in educational data sets. The empirical result shows that (1) the proposed Flat-OCC model can classify and identify the emotion of education public opinion data well; and (2) for the recognition of educational emotion classification, the categorization accuracy of the Flat-OCC+EMO-CBOW model is significantly higher than that of a single Flat-OCC model, which reveals that the emotional-pack-based model we propose can enhance our benchmark model.

## Background

Since 2003, researchers have paid more and more attention to emotion analysis in the field of Natural Language Processing (NLP). Text information is usually divided into two categories: one is factual information that only contains an objective expression of entities or events, and the other is to express the author's actual feelings and opinions about entities or events. Sentiment analysis takes the concept of emotion as personal information and explores a person's feelings and opinions about a specific topic (Turney, 2002). Opinions play an essential role in human activities. Our views on an unavoidable reality and the choices we make are likely to be influenced by others. Therefore, sentiment analysis of Education public opinion text has essential application value in many fields. Deep learning has shown significant advantages in text processing (Gupta et al., 2021; Malviya et al., 2022). In the early stage, Kim and Hovy (2006) used the dictionary to classify emotions from the set of seed emotion words collected by hand. Tan et al. (2009) proposed a sentiment classification system based on semi-supervised feature extraction. Johnson (2017) and Rajasegaran et al. (2019) used the CNN-based model to extract features and classify emotions from texts. Deep learning models have also been well applied in sentiment analysis (Gupta et al., 2021; India et al., 2022). Meanwhile, Deep learning has also had excellent applications in the field of Education (Debnath et al., 2022).

Based on the Flat-OCC model, this paper first proposes a deep learning method, which is highly dependent on the annotation process of tagging corpus. At the same time, it summarizes the similarity of similar emotional corpora based on structural model, and describes the simple rules that computer language can realize through formal language. This method can also be used to predict the annotation corpus of experiments; the method is used. Based on this, the author further abstracts and improves the rules, proposes the method of keyword sequence tagging, converts the matching of rules into the search for crucial attribute tags, and judges the possibility of containing some emotion in the test corpus according to the score formula.

This paper includes phrase- and sentence-level research. Categories cover 12 different emotions. Statistical and rule-based methods are considered. Major contributions include:

1. It enriches thc processing and emotion computing and is no longer limited to the classification of positive and negative semantic orientation. It explores multi-category and deep emotion detection by combining the most widely recognized multi-category emotion description model.
2. A Flat-OCC model is proposed to induce 12 emotion categories. The connotations and denotations of each defined emotion are given, and the elements in each emotion judgment rule are further clarified, making it easier to use formal language descriptions for each emotion.
3. Based on the Flat-OCC model, two implementation methods are proposed. The effects of heuristic rules and keyword sequence tags on multi-category emotion recognition are analyzed and compared through experimental verification, as well as their advantages and disadvantages and application environments.
4. This paper proposes a word vector model emo-CBOW which integrates emoticons.

## Summary of existing studies

Nasukawa put forward the concept of Sentiment Analysis in 2003, after which many researchers conducted in-depth studies on the subject. There are three existing research methods: those based on dictionaries and rules, those based on traditional machine learning, and those based on deep learning, which are getting more attention (Nasukawa and Yi, 2003).

**Figure 1:**
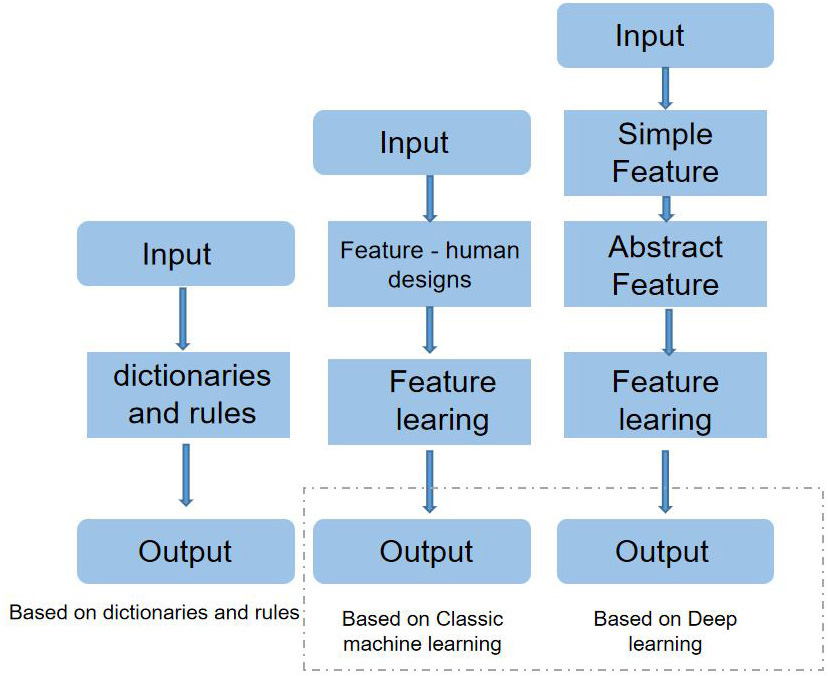
High-level flow chart of the three methods used for emotion analysis.

Figure 1 is the processing flow chart of the three methods; the shaded box represents the components that can be learned from the data. It can be seen from the block diagram that both the methods based on dictionaries and rules and the methods based on traditional machine learning inevitably design the feature manually, and the effect of this step is often not as good as the feature automatically extracted by the deep learning network. In deep learning-based methods, simple features are generally represented by word vectors, while deep learning network structures extract more abstract features.

In the early stage of text emotion analysis, the method is mainly based on a dictionary and rules, which calculates the emotion score of the text by matching the pre-constructed emotion dictionary to the syntactic rules of sentence patterns. Kim (2006) took the existing emotion dictionary as a matching library. They calculated the emotion score after traversing the emotion dictionary of the sentence to be analyzed to determine the emotional polarity of the text. Tang et al. (2014a) constructed an emotion dictionary containing 178,781 positive and 168,845 negative words using the representation learning method. The emotion analysis based on the dictionary achieved an accuracy rate of nearly 86% in the SemEval2013 dataset. Although the method based on an emotion dictionary is not complicated, the language expression forms are changeable, and the constructed dictionary and rules cannot comprehensively cover all kinds of situations. They cannot be used universally for dictionaries and rules constructed in different languages.

The machine learning-based method inputs the hand-selected effective text representation features into an effective classifier to solve the sentiment classification task in sentiment analysis. This method overcomes the limitations of dictionary and rules-based methods, which are limited by dictionary size and rule comprehensiveness. Feature engineering is the core of machine learning-based methods. Most researchers improve feature engineering to improve the accuracy of emotion analysis tasks. Pang et al. (2002) combined the word bag with speech and other different combination features on the classifiers, then input them into three standard machine learning classifiers for experiments, and compared the performance of different combination features on the classifiers. Abbasi et al. (2008) proposed that syntactic features such as N-Gram, Part-Of-Speech (POS), and punctuation should be combined with structural features such as word length and word format. The feature selection method uses the Information Gain (IG) value. Wilson et al. (2009) combined syntactic features such as words, negative words, emotional modification, and emotional transfer for emotion analysis and obtained good experimental results. In literature, TF-IDF, IG, MI, and Chi-square statistics were selected under a naive Bayesian classifier to conduct comparative experiments. The effects of these features in different Chinese sentiment analysis tasks were investigated. A small number of researchers have made improvements to classifiers. Wang and Manning (2012) proposed a combined model of SVM and NB based on the advantages and disadvantages of SVM and NB, and the experimental results of the combined model were superior to other models in multiple datasets. The accuracy of the emotion analysis method based on machine learning has dramatically improved compared with that based on the emotion dictionary. However, the performance of this method is closely related to the quality of feature engineering, which not only costs a lot of manpower, energy and material resources but also has the problems of incomplete artificial feature mining and poor system migration.

Using deep learning to solve emotion analysis tasks has attracted wide attention from researchers in recent years. This method can overcome the problem that machine learning-based methods need artificial feature engineering, can automatically extract compelling features, and show a significant improvement in performance compared with methods based on emotion dictionaries and machine learning. There are two critical problems with this approach:

1) Word vector

Learning contains contextual semantic information in a massive corpus, based on continuous term vectors plays a vital role in the analysis of deep learning emotions, and the methods of obtaining word vectors mainly include two categories: using the method of unsupervised intrinsic semantic information of the text clustering statistics model and neural network training to get the word vector model.

In the first method, the method based on LDA is more commonly used (Blei et al., 2001). The LDA method is used to learn the pre-set number of topics, and the probability distribution of each word under different topics is calculated in a large corpus, which is used as the word vector of the word. In the second method, words in the text are often mapped into multidimensional vectors by the one-hot representation method in the early stage. However, this method only converts words into symbols and fails to fully reflect the relationships and deep meanings between contextual words (Wang and Manning, 2012). In 2003, Bengio et al. constructed a statistical Language Model (Neural Network Language Model (NNLM) by using a Neural Network and extracted the by-products generated in the process of training the probability model as word vectors (Bengio et al., 2006). The word vectors trained by this model capture the location and semantic information between words.

In 2008, Collobert and Weston proposed the C&W model to improve the efficiency of word vector training, which can directly learn word vectors (Collobert and Weston, 2008). In 2013, Mikolov et al. simplified and retained the core part based on the NNLM model and C&W model and took the word in the middle of a paragraph of text as the target word and the words around the middle word of a paragraph of text as the target, and proposed the Word2VEC framework (Mikolov et al., 2013). The framework consists of two Continuous Bag-of-word (CBOW) models and Skip-Gram models, which use optimization strategies to reduce the time cost of word vector training and are popular word vector models in NLP tasks.

The word vector training method mentioned above is learned from unsupervised data. It only obtains semantic relation and grammatical information of context but fails to fully use emotional information, so its performance in emotion analysis tasks is not ideal. Therefore, combined with the characteristics of the emotion analysis task, researchers use supervised text to re-learn the conventional word vector model so that the trained word vector contains emotional information. Maas et al. (2011) proposed a probabilistic model (Senti-LDA) based on LDA that contains both semantic information and emotional information, and the accuracy of the word vector under the model is higher than that trained by the LDA model in the sentiment analysis dataset. Tang et al. (2014b) improved the loss function of the C&W model by adding supervised emotional information to learn the word vector containing emotional information. Zhang and Man (2016) improved the loss function of CBOW and Skip-Gram models and also integrated supervised emotional information to obtain an emotion word vector. However, these word vector models require the construction of many data sets containing emotional labels in advance, which is labor-intensive.

2) Deep learning model to extract high-level text features

In early studies, Parse Tree features containing syntactic and semantic information were often used to input the original words into the neural network for learning to infer the emotional components better. However, in recent studies, deep learning networks have become increasingly popular. They do not require analytic trees as input but instead use word vectors containing semantic and syntactic information trained from vast amounts of unsupervised data. In addition, deep learning networks can further learn texts' context-dependence and syntactic features. Socher et al. (2011) first proposed a semi-supervised Recursive Autoencoders Network (RAE) for sentence-level sentiment classification, which can obtain the vectorized representation of text dimensionality reduction. Kalchbrenner et al. (2014) proposed a Dynamic CNN network for sentence semantic modeling, which uses a Dynamic K-max Pooling operation as a nonlinear down-sampling function, and features obtained from the network can capture the relationship between words. Subsequently, Kim (2014) also proposed to use the CNN network for sentence-level sentiment classification. According to the input of the word vector, the input of the word vector is classified into CNN-rand (the word vector is initialized), CNN-static (the word vector is pre-trained and fixed), CNN-non-static (the word vector is pre-trained and adjustable), and CNN-rand (the word vector is initialized and fixed) CNN-multichannel (two sets of word vectors are used as input at the same time). Through the experimental results of these groups, we can see the importance of pre-trained word vectors and the advantages of the CNN network over the RAE network and classical machine learning. Subsequently, Xin et al. (2015) used the LSTM network to conduct sentiment analysis on Twitter, simulated the interaction between constructed words, and performed the multiplication operation between word vectors through gate structure to provide greater flexibility. Compared with the additive results in a simple RNN network, this network structure produced better-combined results.

In the field of cognitive psychology there have been long-term and in-depth studies on emotion (Frijda, 1986; Scherer et al., 2001). Related theories and models explain the causes of emotions and their effects on cognition and behavior. This theory provides a conditional model in which different emotions appear as corresponding results according to corresponding conditions. Within the literature related to emotion in cognitive psychology, cognitive assessment theory is the most comprehensively studied and mature. In particular, the OCC (Ortony et al., 1988) model proposed by Ortony, Clore, and Collins is a widely used model. Aiming at the emotion modeling of a computing system, the OCC model believes that specific emotions are formed under the attention of events, people and objects, and generate specific emotions through potential cognitive structures. The affective dimensions of these cognitive structures include essential variables, fan-worthy or blame-worthy. From an implementation point of view, the OCC emotion pattern can be thought of as a set of inference rules leading to the final emotion type.

## Word vector model incorporating emoticons

By using the deep learning sentiment analysis model, the first problem is how to get the words that need to be solved which the computer can recognize the digital form. The word vector model based on neural network can learn low-dimensional vectorization in massive unsupervised data. The word vector model is a representation method of excellent word performance, it can be directly used as a unique feature training emotion analysis model. However, previous word vector models only learn syntactic and semantic information and lack critical emotional information in emotion analysis tasks. Simple text expressions lack facial expressions and body language in face-to-face communication, so readers cannot perceive the true emotions of the sender. In microblogging, emoticons have a powerful expressive force, their connotations and functions are very rich. In addition emoticons in microblogging make expressions clearer and help the receiver to understand better. Microblog in this emoticons in this sentiment analysis has an essential effect to the cmmunication promotion performance. Therefore, based on the CBOW word vector model, a word vector model integrating emoticons EMO-CBOW is proposed in this article, which takes advantage of the more explicit emotional information contained in emoticons in microblog text. The word vector model was applied to the emotion analysis task, and the experimental results showed that the word vector model is better than the contrast model.

### Word vector model integrating emoticons (EMO-CBOW)

In this article, EMO-CBOW, the word vector model of emoji fusion consisted of three steps: constructing an emoji dictionary, word vector training, and word vector calculation of emoji fusion. Firstly, statistics were gathered on the use of emoticons in a large number of microblog texts, and the emoticons were sorted according to their use frequency and stored in the emoticons dictionary. Then, word vectors were trained in the corpus using the CBOW model. The corresponding word vectors of all emoticons in the emoticons dictionary were found to form the emoji representation matrix. Each word vector trained by the CBOW model is related to the expression matrix of emoticons, and the word vector model EMO-CBOW integrating emoticons is finally obtained. The detailed implementation methods of each step are described below.

#### Building an emoji dictionary

**Figure 2:**
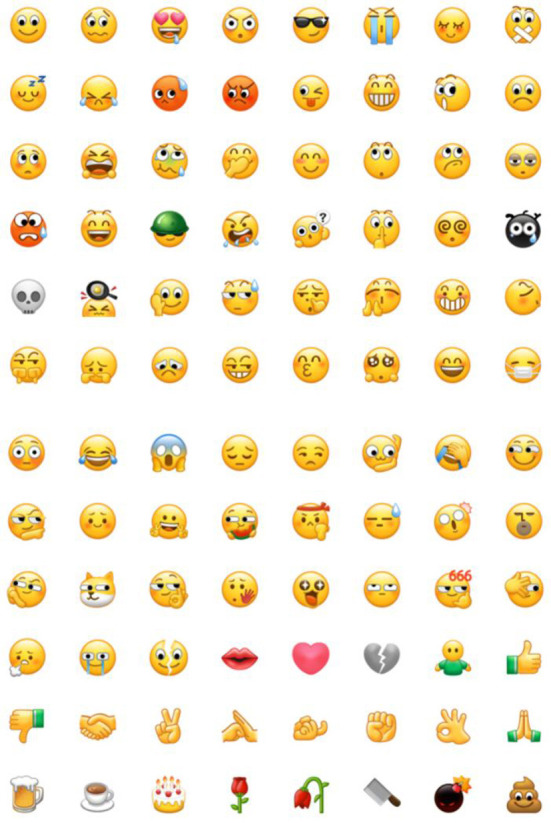
The Emoji in education data.

In our educational database (we collected curriculum review data on the evaluation of teachers and students), emoticons are displayed as shown in Figure 2, but in the text are expressed as the form of “[XX]”. In the statistical corpus of the use of 500,000 educational emoticons, the corpus of emoticons totals more than 10,000 times. It can be seen that it is quite common for students to express their opinions with the help of emoticons when posting comments. This article is sorted according to the frequency of occurrence of emoticons in the whole corpus, and saved in this order as a dictionary of emoticons.

#### Word vector training

In the sentiment analysis of the education public opinion text, the words that comprise the education public opinion text contain essential information from the education public opinion publisher. In this paper, words are regarded as the smallest unit to analyze sentence emotional information; that is, sentences are represented as the splicing of word sequences. Before splicing, words need to be represented as corresponding multidimensional vectors. Two model structures of Skip-gram and CBOW for word vector training based on the neural network have been briefly introduced. In the CBOW model, the context word *context*(*w*) of the center word *w* is used to predict *w* under a given window length; that is, for *context*(*w*), except for the positive sample of the center word *w*, all other words in the dataset are negative samples. Let the negative sample set of *context*(*w*) be *NEG*(*w*), and the label *L*^*w*^(*w*′) of any word *w* in the dataset be:


(1) $${\text{L}}^{\text{w}}\left({\text{w}}^{\prime }\right)=\{\begin{array}{cc} 1,\; & \;\\ \; & w^{\prime }=w\\ 0,\; & \;\\ \; & {\text{w}}^{\prime }\neq \text{w} \end{array}$$

For a positive sample (*context* (), *w*), maximize


(2) $$\begin{array}{l} \text{g}\left(\text{w}\right)=\prod_{\text{u}=\left\{\text{w}\right\}\text{U NEG}\left(\text{w}\right)}p\left(\text{u}|\text{context}\left(\text{w}\right)\right) \end{array}$$

Among them,


(3) $$\text{p}\left(\text{u|context}\left(\text{w}\right)\right)=\{\begin{array}{cc} \sigma (X_{w}^{T}\theta^{u}),\; & \;\\ \; & {\text{L}}^{\text{w}}(\text{u})=1\\ 1-\sigma ({\text{X}}_{\text{w}}^{\text{T}}\theta^{\text{u}}),\; & \;\\ \; & {\text{L}}^{\text{w}}(\text{u})=0 \end{array}$$

Where *X*_*w*_ is the sum of all word vectors in *context*(*w*), *θ*^*u*^ ∈ *R*^*n*^ is the trainable parameter vector of *u* in the model, and $\sigma \left(X_{w}^{T}\theta^{u}\right)$ is the probability of predicting the center word *w* when the context is *context*(*w*). The training goal of this model is to maximize *g*(*w*), and the Formula above is created to obtain


(4) $$\text{g}\left(\text{w}\right)=\sigma (X_{w}^{T}\theta^{u})\prod_{\text{u}\in \text{NEG}(\text{w})}\left[1-\sigma ({\text{X}}_{\text{w}}^{\text{T}}\theta^{\text{u}})\right]$$

That is to increase the probability that the selected word is a positive sample and decrease the probability that the selected word is a negative sample. For a given corpus W, the overall optimization objective function of the model can be obtained.


(5) $$\begin{array}{l} \text{F}=\prod_{\text{w}\in \text{W}}\text{g}\left(\text{w}\right) \end{array}$$

After differentiating the objective function F, the stochastic gradient descent method is used to update the model parameters, and finally, the word vector is obtained. The saved word vector format is that each word corresponds to a fixed dimension vector.

#### Word vector calculation integrating emoticons

**Figure 3:**
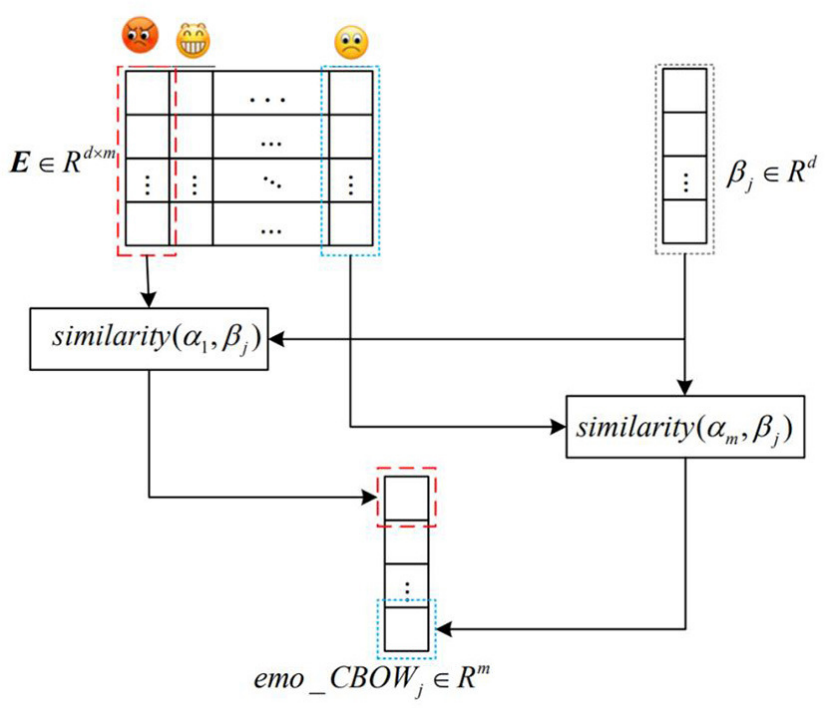
Word vector construction diagram integrating emoticons.

The word vector of dimension *d* corresponding to emoticons with the highest frequency of *m* in the word vector model was taken out for horizontal splicing to form the affective symbol space matrix $E=\left[\alpha_{1},\alpha_{2},\cdots \;,\alpha_{m}\right]\in R^{d\times m}$, in which the values of *m* and *d* were selected through experiments. Traversal word vector model; Each term corresponds to a vector space and emotion symbol matrix according to formula (6), do arithmetic, and save the result as a fusion emo-CBOW word vector model of emoticons. The term vector of each dimension represents a dimension value similar to m emoticons and similar to some emoticons. On the contrary, the more obvious the semantic difference of emoji, the closer the calculated value is to 1. Conversely, the more significant the semantic difference from an emoji, the closer the calculated value is to −1. Therefore, the word vector model in this chapter endows the CBOW model with more abundant emotional information. The build schematic is shown in Figure 3.


(6) $${\text{emo}}_{{\text{CBOW}}_{\text{ji}}}=\cos\left(\alpha_{\text{i}},\beta_{\text{j}}\right)=\frac{\alpha_{\text{i}}^{\text{T}},\beta_{\text{j}}}{\left\lceil \alpha_{\text{i}}\right\rceil \left|\beta_{\text{j}}\right|}\text{ i}\in \left[\text{1},\text{m}\right],\text{ j}\in \left[\text{1},\text{n}\right]$$

Where α is the emoji word vector, β is the original word vector, *m* is the number of emoticons in the emoticon matrix, *n* is the total number of words in the original word vector model, and the dimension of the word vector transformed into EMO-CBOW model is *m*.

### Algorithm flow

Input: Education public opinion corpus for word vector training *D*_*word*_, annotated corpus for emotion analysis model training *D*_*rain*_, test set corpus for emotion analysis model performance testing *D*_*test*_. The emoticons appearing in the corpus are counted and sorted according to occurrence frequency and saved as emoticons dictionary *D*_*emo*_.

Output: Emotion category of Education public opinion text *l*.

**Phase 1**. Train word vectors

Step1 Preprocess the input corpus *D*_*word*_, *D*_*rain*_ and *D*_*etest*_, and then use the Python word segmentation tool jieba to segment the microblog text.

Step2 Initialize parameters of the CBOW model.

Step3 For each Education public opinion text *s* ∈ *D*_*word*_

Step4 For each word *w*_*i*_ ∈ *s*

Step5 Calculate the collinear probability of k words in the context of *w*_*i*_ according to CBOW model architecture.

Step6 End For

Step7 Maximize the objective function

Step8 End For

Step9 Save all the semantic word vector *X*_*CBOW*_, and the dimension of each word vector is *d*.

Step10 For each emoji *w*_*j*_ ∈ *D*_*emo*_ *AND w*_*j*_ ∈ *X*_*CBOW*_

Step11 Take out the word vector of *w*_*j*_ and splice it into line

Step12 End For

Step13 Save the emoji word vector matrix *X*_*emo*_

Step14 For each word *w*_*j*_ ∈ *X*_*CBOW*_

Step15 Take out the word vector β_*i*_ and the former *m*-dimension of the emoji word vector matrix *X*_*emo*_ for relevant calculation.

Step16 Save all the word vector *X*_*emo*_*CBOW*_ that integrates emoticons, and the dimension of the word vector is *m*.

**Phase 2**. Train the emotion analysis model

Step1 Initialize parameters of the convolutional neural network

Step2 For each training text *s*_*i*_ ∈ *D*_*train*_

Step3 Find the word vectors of all words from the word vector *X*_*emo*_*CBOW*_o that integrates emotional symbols to form the text representation matrix $X_{i}=\left[x_{1},x_{2},\cdots \;,X_{n}\right]\in R^{n\times m}$

Step4 Convolved multiple different convolution kernels with the length *m* of the convolution window of 3, 4, and 5, respectively with the text representation matrix, and obtained the feature vector *C* = [*c*_1_, *c*_2_, ⋯ , *c*_*n*−*m*+1_] according to the complete pooling rule.

Step5 Input feature vector *C* into softmax function to obtain the category label *l* of the input sample

Step6 Update model parameters by batch gradient descent method

Step7 End For

Step8 Save the CNN model parameters obtained by training.

**Phase 3**. Test the emotion analysis model

Step1 For each test, text *s*_*j*_ ∈ *D*_*test*_

Step2 Find the word direction of all words in *X*_*emo*_*CBOW*_ and group them into the text to represent the moment matrix $X_{i}=\left[x_{1},x_{2},\cdots \;,X_{n}\right]\in R^{n\times m}$

Step3 Load CNN model parameters, input text representation matrix, and obtain emotion category labels of test samples

Step4 End For.

## The realization of categorization of education public opinion sentiment based on flat-OCC

### System overview

There have been many studies on emotional sensation in psychology and cognitive science. The OCC model is an emotional sensation model developed based on cognitive psychological research and based on computer reality. OCC mode is recognized as a feeling from people's recognition and evaluation of the situation formed by events, agents, and objects. The basic principle of OCC theory is to divide people's responses to specific situations into positive or negative categories according to a set of rating scales and then determine the corresponding emotion categories according to the different value combinations of the rating scales.

Combined with the characteristics of the Chinese Education public opinion corpus, this paper constructs a flat-OCC model in which 14 emotions are grouped. They are blessing, jealousy, thankfulness, sympathy, hope, worry, joy, sadness, pride, shame, admiration, contempt, pleasure, and disgust.

Firstly, some rules are formulated to screen out candidate corpora that may contain emotion. The preprocessing is based on the selected corpus, and the self-defined seed words are used as the dictionary during word segmentation. Meanwhile, a series of rules are established to filter out some possible interfering contents according to the composition characteristics of Education public opinion.

At the same time, to make the annotation of multi-category emotional corpus easier, a multi-category annotation module is developed, which realizes the essential information visualization function and facilitates the adjustment of annotation strategy at any time. Some popular open source tools and frameworks are used to annotate new corpus. The front end of the system mainly uses the Jade template framework and Stylus style preprocessing framework to build the necessary user interface. At the same time, the back-end services run on personal VPS virtual machine servers, which support the IP dual-stack protocol and ensure that the service can be used with ordinary broadband Internet and education networks. The server framework uses NodeJS-based Express framework, which can quickly define and implement the corresponding interface of annotated corpus and experimental test results using RESTFUL style. The open source document database MongoDB is used to store the final data store.

Finally, the corresponding meanings and expressions of each emotion in Flat-OCC in the Education public opinion corpus are clarified through the labeling process analysis. Two methods to realize the conditional rules in Flat-OCC are obtained: the heuristic rule method and the keyword sequence tag-based method.

### Preprocessing module of corpus

The preprocessing module of the experimental corpus encapsulates the natural language primary tool interface of HIT Language cloud. The interface uses the form of RESTful API and supports both GET and POST. By using this essential tool, the experiment can provide tools to carry out such tasks as word segmentation and semantic role labeling on the corpus and obtain structured representation information of the corpus for use in subsequent methods and experiments.

In addition to a series of processing on the Education public opinion corpus mentioned above, such as word segmentation and semantic role labeling, it is also necessary to clean and filter the Education public opinion corpus. As there are many useless elements in the Education public opinion corpus, these elements are not helpful for follow-up research and may cause other problems. It is also necessary to remove and filter the above useless interference information. In order to achieve this goal, a series of filtering rules should be established according to the characteristics of Education public opinion.

### Corpus annotation module

Since manual labeling is time-consuming and labor-intensive, especially for 12 categories of emotions, it is generally necessary to fully understand multiple categories' connotations and denotation of emotion concepts to complete the labeling task. If there are many categories, it is possible that annotators will put too much energy into classifying the current corpus according to different categories of concepts, which will slow down the speed of annotation and confuse the annotators with multi-category discrimination.

In order to simplify the workload of manual annotation and check the effect of the model at any time, an easy-to-use visual system for corpus annotation and analysis was built. The system tries to reduce the workload and difficulty of manual annotation to the greatest extent; it only needs to determine whether a specific category of emotion is included in the annotation. That is to say; the annotator only annotates the candidate corpus of one particular emotion at a time. The annotator is required only to have a clear concept of this emotion instead of covering all kinds of emotions. At the same time, the system can also achieve the classification of a corpus by using the heuristic rules in the following section, and it is relatively easy to call the corpus analysis program to analyze and judge a new corpus according to the current language model as well. Annotate the corpus and, expand the corpus, continuously improve the quality of tags and rules. The updating of the critical information in the newly annotated corpus is completed automatically by the computer; that is, starting from the initial seed words, the newly annotated corpus is analyzed by essential tools to obtain the corresponding object representation, including the critical basic information such as word segmentation, part of speech tagging and semantic dependency relationship. The key attributes of emotions of the same kind, such as part of speech, semantic function, interdependence, are compared and counted horizontally in the new corpus, and the number of occurrences of new words is extracted from the corpus. When the conditions are appropriate and the number of occurrences reaches a certain number, words are added to improve the seed of new words, so as to expand the seed by using the iterative thesaurus. Table 1 is the pseudo-code of the Haskell class describing the process of expanding the thesaurus above

**Table 1:** Initiation rules modeling method process.

type alias word =
{player: string, pos: String}
type alias words =
[word]
Seed:: words −> words
Seed init =
1et
– condition:: words −> Bool
condition ss =
if fulfill ss then
True
else
False
– new:: Signal words
new = tagged
in
fold conditions init new

The labeling system design's original purpose is to simplify the manual annotation workload. By selecting an emotion type, the candidate corpus of this emotion type is selected from the database, and then the corpus of the candidate corpus is marked. The marked options are “yes,” “no,” and “skip”. “Yes” means that the current corpus contains the emotion type being marked. “No” means that the current corpus does not contain the emotion type being marked. “Skip” can temporarily ignore the current corpus and label some uncertain corpus later. Interface “/data/emotion/?{type}” is an emotion class type that needs to be marked before selecting the appropriate one. Every time it selects an emotion class type, it will automatically ask interface “/data/emotion/?{type}”, which will return “/data/emotion/?{type}” candidate corpus with emotion type “type”. Then one can select “Yes” or “No”. Interface “/data/emotion/?{type}” will be called to annotate the corpus. After annotation, a new candidate corpus will be automatically obtained for annotation. Table 2 shows the marking operation of the interface.

**Table 2:** Marking operation of the interface.

**Start annotating tasks of the type class**	**“get:/data/emotion/? {type}”**
Gets the unannotated corpus of the type class	“get:/data/getit/? {type}”
Emotional example sentences labeled as type	“post:/data/tagit/? {type}”
Emotion example marked as opposite to the current type	“post:/data/taganother/? {type}”
Emotion example marked as other emotional examples of type	“post:/data/pickone/? {type}”

However, it was found that due to the selection criteria of candidate corpora, there are many similar but opposite types of corpora and some other types of corpora in each type of candidate corpora. In the database, all the corpus is put into a document, and each corpus has its single id and a field to indicate whether or not it has been labeled. Only the id of the corpus is recorded in the corpus documents determined by each emotion type. In the original annotation method, only one corpus is allowed to be marked once. Once marked “no”, even if the corpus contains other types of emotion, it will be discarded. Therefore, interface “/data/taganother/? {type}” and Tag Another option is added to indicate that the current corpus is an emotion type relative to the previous emotion type. In order not to discard potentially valuable corpus, the “/data/pick one/? {type}” interface and the PickOne option select an emotional type of attribution to annotate the current corpus. “Pick one” can fully achieve the effect and function of “TagAnother”, because “TagAnother” has more scenes and can quickly mark the corresponding type of corpus without selecting the type and then labeling.

The simultaneous summary corpus of annotated corpus appears most frequently. It is most likely to be used as keywords in critical sequences of different emotions in the Flat-OCC model, and related words or phrases are included in the emotion keyword database. The above 12 categories of emotions are classified by the positive and negative aspects of their emotional attributes. The emotions with the same reference conditions include both positive and negative emotions. For example, the emotions concerned about the event have “hope” (positive) and “worry” (negative). The two kinds of emotions with opposite polarity of the same condition can be considered the same in the type of key emotion sequence.

The annotated corpus module also combines the training and testing functions of the corpus and gradually improves the effect by iteratively adding new manually annotated corpus. Word segmentation and part-of-speech tagging are used for a newly annotated corpus to extract keyword sequences and expand the probability model. Firstly, the new microblog corpus labeled with emotion type label is sectioned. Since all the service interface codes are in the same space, we can directly use the internally encapsulated method of calling the language cloud interface to directly send HTTP Post request to it, carrying the request load as corpus text to be marked, instead of sending an extra HTTP request to the local address. Then, part-of-speech tagging is performed on the corpus text after word segmentation. The steps of part-of-speech tagging are similar to word segmentation, and the provided methods are also used.

### Simulation method of heuristic rule

Processing corpus text using the essential tools mentioned in the previous section can yield a structured representation of the corpus that can be processed as an object. The idea of the heuristic rule simulation method is that according to the description of each emotion triggering condition in the Flat-OCC model, the most direct way to identify multiple emotions is to obtain the emotion category according to the value of the judgment elements in the condition in the current corpus.

We can establish an immediate recognition and matching system according to the corresponding rules. The positions of capitalized words in the rules represent the keywords contained in this type of emotion and the semantic roles in the analysis of semantic dependency relations, in which the keywords are initially selected and recorded by the vital seed words in the initial corpus tagging process.

When we annotate the corpus, we can also preliminarily predict the emotion types of the new corpus as preliminary test data and observe the changes in the accuracy of prediction in the process of the expansion of the seed thesaurus. The prediction method is to screen the key attributes in the new corpus, which can be divided into strict judgment and loose judgment. Heuristic rules for each emotion are described in Table 3; rules are connected by logical words “and” and “or”, among which different parts are connected by and are critical elements of this affective rule in Falt-OCC. For example, the rule of the worry emotion category is “CORPUS contains FUTURE and AO contains self and(CEN_V or Al)is NEGATIVE”, among which elements are “CORPUS contains FUTURE”, “A0 contains self,” “(CEN_V or Al)is NEGATIVE,” three key elements, in which the strict judgment method of the above summary of each type of emotional rules of the key elements all need to contain, for example, the above three elements need to contain; For example, the above three elements need to have “CORPUS contains FUTURE” and “(CEN_V or A1)is NEGATIVE”. At any time, the differences between the predicted and manual annotation results of the two types of methods for a new corpus are counted as preliminary test results.

**Table 3:** Rules of 13 types of emotion models.

**Gratitude**	**A0 contains SELF, and (CEN_V or A1) is POSITIVE, and PRP contains OTHER**
Jealousy	A0 contains OTHER, and (CEN_V or A1) is POSITIVE + A0 contains SELF, and (CEN_V or A1) is NEGATIVE.
Thankfulness	A0 contains [OTHER and SELF] and (ADV of OTHER) is NEGATIVE, and (A1 of SELF) is NEGATIVE
Sympathy	A0 contains [OTHER and SELF] and (A1 of OTHER) is NEGATIVE, and (A1 of SELF) is NEGATIVE
Hope	CORPUS contains FUTURE and A0 contains self, and (CEN_V or A1) is POSITIVE
Worry	CORPUS contains FUTURE and A0 contains self, and (CEN_V or A1) is NEGATIVE
Joy	A0 contains self, and (CEN_V or A1) is POSITIVE
Sadness	A0 contains self, and (CEN_V or A1) is NEGATIVE
Pride	A0 contains SELF and A1 contains SELF, and CEN_V is (SYNONYM of PRAISE)
Shame	(A0 or A1) contains SELF and or (SYNONYM of PRAISE and ADV of CEN_V is NEGATIVE)
Admiration	A0 contains OTHER, and A1 contains (SYNONYM of PRAISE)
Contempt	A0 contains OTHER, and A1 contains (SYNONYM of BLAME)
Disgust	A0 contains OBJECT, and CEN_V or A1 or (ADV of CEN_V) is NEGATIVE, and CEN_V contains (SYNONYM of HATE)

### Keyword sequence tag method

Through the above heuristic summary of some rules of the OCC model, we try to apply the rules preliminarily while annotating the corpus. However, we can see that the intuitive and straightforward application of rules is far from enough to achieve the desired effect. Therefore, the rule model should be further abstracted and simplified, and the ultimate goal should be to improve judgment accuracy.

Through experiments, it is found that the effect of the loose judgment method described in the above section is slightly better than that of the strict judgment method. In addition, while maintaining the evaluation condition information in the OCC model, conditions can be formalized into the combination of keywords, that is, the keyword sequence. Each keyword belongs to a critical attribute tag. Multiple keywords can belong to the same key attribute tag. Therefore, by using the keyword tag sequence of abstract rules, emotional keyword tags are organized in the form of key values, that is, they can be used for direct export modules of programs. The key attribute tags corresponding to each emotion are as follows, and each key attribute contains several keywords at the same time, thus continuously expanding keywords from the seed thesaurus. Table 4 shows the key tags sequence of 12 emotion types.

**Table 4:** Key tags sequence of 12 emotion types.

**Hope**	**[“self,” “prospect,” “hope,” “positive”]**
Worry	[“self,” “prospect,” “fear,” “negative”]
Joy	[“self,” “positive”]
Sadness	[“self,” “negative”]
Blessing	[“other,” “desire,” “self,” “auxiliary,” “positive,” “happy”]
Jealousy	[“other,” “desire,” “self,” “auxiliary,” “negative,” “resentment”]
Thankful	[“other,” “undesire,” “self,” “gloating,” “positive”]
Sympathy	[“other,” “undesire,” “self,” “pity,” “negative”]
Pride	[“self,” “praiseworthy,” “proud,” “positive”]
Shame	[“self,” “blameworthy,” “shame,” “negative”]
Admiration	[“other,” “praiseworthy,” “self,” “appreciation”]
Contempt	[“other,” “blameworthy,” “self,” “reproach”]
Disgust	[“obj,” “hate,” “cons”]

It also includes common keyword tags, as shown in Table 5.

**Table 5:** Analysis of typical example sentences for each type of emotion.

**Positive**	**There are 33 universal positive words, such as excellent, happy**
Negative	There are 34 universal negative words, such as mad, wronged, sad
Yes	There are 12 positive words, such as: yes
No	There are 14 positive words, such as: no, don't
Self	There are 31 words to represent oneself, such as I, we, and myself
Other	There are 31 words to represent other people, such as another person, she, UserName
Praise	The number of praiseworthy actions is 27
Blame	The number of blameworthy actions is 34

The above sequences of critical tags are not a set of key sequences, and the sequences' orders may also express specific meanings. The closer the sequence of tags is to the judgment order of Falt-OCC rules, the more likely the corpus containing this sequence will contain this kind of emotion. For example, the critical tag sequence of a certain kind of emotion corpus is: [other desire self pos]; this corpus is very likely to contain the “blessing” type of emotion; each label has its weight according to the importance of each label. Therefore, according to the keyword tag sequence model, we obtain the score formula 1 of a particular emotion based on the object representation of each corpus after word segmentation.


(7) $$\begin{array}{l} \text{P}\left(\text{emotion},\text{ corpus}\right)\\ =\frac{\text{richness}{\left(\text{emotion},\text{corpus}\right)}^{*}\sum_{\text{i=1}}^{\text{n}}{\text{weight}}_{\text{i}}^{*}\text{appear}(\text{emotion},\;{\text{tag}}_{\text{i}})}{\left(1\text{−}\alpha \right)*\text{tightness}(\text{emotion},\text{corpus})} \end{array}$$

Formula *P* accepts two parameters. Namely, emotion judged and corpus judged. The scoring Formula consists of several parts: *keywords*, *tightness*, and *richness*. The explanations for each part are as follows: *keywords* represent the object representation of the critical word sequence after word segmentation in the corpus, and *tagSeq* is the function to analyze the corpus. The function group comprises key phrase regular expressions containing various category tags.


(8) $$\begin{array}{l} \text{keywords}\left(\text{e},\text{c}\right)=\text{tagSeq}\left(\text{e},\text{c}\right) \end{array}$$

*Tightness* represents the compactness of a corpus. Since Education public opinion corpus is long and generally composed of multiple sentences, we hope to find out the part that contains the most concentrated emotional information in the sentence. Find the difference between the last and the first position of all the keywords in the corpus.


(9) $$\begin{array}{l} \text{tightness}\left(\text{e},\text{c}\right)=\frac{\text{last}\left(\text{keywords}\left(\text{e}\right)\right)-\text{head}\left(\text{keywords}\left(\text{e}\right)\right)}{\text{length}\left(\text{c}\right)} \end{array}$$

*uniqKey* represents the sequence of keywords after the duplicate keywords are removed, where *unique* is a function of de-duplication.


(10) $$\begin{array}{l} \text{uniqKey}\left(\text{e},\text{c}\right)=\text{unique}\left(\text{keywords}\left(\text{e},\text{c}\right)\right) \end{array}$$

Where *richness* represents the indicator of the richness of keywords in the corpus, the more times different keywords appear in the key sequence of a certain emotion, the greater the value of *richness* will be.


(11) $$\begin{array}{l} \text{richness}\left(,\text{c}\right)=\frac{\text{length}\left(\text{uniqKey}\left(\text{e},\text{c}\right)\right)}{\text{length}\left(\text{keywords}\left(\text{e},\text{c}\right)\right)} \end{array}$$

*weight* represents the weight of each label, *appear* is a method that can return whether the key label appears in a certain emotion category, and *tag*_*i*_ represents the key label identified by the system.

Formula (7) calculates the score of a corpus containing certain emotions. As the expression mode of the Education public opinion corpus is rich and non-standard, the whole Education public opinion is a complete corpus. The relationship between different sentences may not be strictly contextual, and the intention expressed may be different or even opposite. Therefore, the purpose is to find possible passages containing emotions, then calculate the total score of the passages containing emotions, judge the possibility of the passage containing certain emotions, and take the emotion type contained in the highest score paragraph as the emotion type contained in the Education public opinion corpus.

## Experimental results and analysis

### Experimental evaluation method

In the heuristic rule simulation experiment, only the changes in accuracy in different stages were observed. The effective keyword tag sequence method uses the F value to evaluate its effect, which is defined in Formula (12). The accuracy rate is the number of correctly classified corpus divided by all the corpus judged by the system to contain emotion. The recall rate is the number of correctly classified emotional corpora divided by the number of annotated corpora containing emotions.


(12) $$\begin{array}{l} \text{F}-\text{score}=\frac{2\times \text{ accuracy rate }\times \text{recall rate}}{\;accuracy\;rate+recall\;rate} \end{array}$$

### Results and analysis

Tables 6, 7 show a confusion matrix for the 12-Class sentiment classification of the proposed model.

**Table 6:** 12-Class sentiment classification confusion matrix (Flat-OCC).

	**Worry**	**Joy**	**Sadness**	**Blessing**	**Jealousy**	**Thankful**	**Sympathy**	**Pride**	**Shame**	**Admiration**	**Contempt**	**Disgust**
Worry	0.81	0.01	0	0.03	0.04	0.01	0.01	0	0.04	0.01	0.04	0
Joy	0	0.83	0.05	0	0	0.01	0	0	0.02	0.05	0	0.04
Sadness	0	0.02	0.65	0.04	0.08	0.04	0.01	0.05	0.05	0.04	0	0.02
Blessing	0.04	0.02	0.05	0.71	0	0.01	0	0.1	0.01	0	0.01	0.05
Jealousy	0.05	0	0.05	0.02	0.61	0.12	0.04	0.01	0.05	0	0.05	0
Thankful	0.04	0	0	0	0.12	0.59	0.06	0.1	0	0.04	0.05	0
Sympathy	0	0.01	0.04	0.01	0.1	0	0.78	0.05	0	0	0.01	0
Pride	0	0	0.02	0.12	0.07	0.04	0	0.71	0.04	0	0	0
Shame	0.01	0.02	0	0.03	0.04	0.01	0.17	0	0.67	0	0.05	0
Admiration	0.04	0.05	0.04	0	0.01	0.01	0.04	0	0.01	0.8	0	0
Contempt	0.01	0.01	0	0	0	0.05	0	0	0.05	0.05	0.83	0
Disgust	0	0.03	0.1	0	0.8	0.03	0.05	0.04	0	0	0	0.67

The score in the list is the highest.

**Table 7:** 12-Class sentiment classification confusion matrix (Flat-OCC+EMO-CBOW).

	**Worry**	**Joy**	**Sadness**	**Blessing**	**Jealousy**	**Thankful**	**Sympathy**	**Pride**	**Shame**	**Admiration**	**Contempt**	**Disgust**
Worry	0.82	0	0	0.03	0.05	0.04	0	0	0.01	0.04	0.01	0
Joy	0.01	0.83	0.02	0.02	0	0	0.01	0	0.02	0.05	0.01	0.03
Sadness	0	0	0.7	0.05	0.05	0	0.04	0.02	0	0.04	0	0.1
Blessing	0.03	0	0.04	0.75	0.02	0	0.01	0.12	0.03	0	0	0
Jealousy	0.01	0.01	0.04	0.01	0.67	0.12	0	0.04	0.01	0.01	0.05	0.03
Thankful	0	0	0.05	0.1	0.01	0.75	0.05	0	0	0	0	0.04
Sympathy	0.04	0.02	0.05	0.01	0.05	0	0.75	0.04	0	0.01	0.03	0
Pride	0	0.02	0.02	0.12	0.07	0.04	0	0.69	0	0.04	0	0
Shame	0.01	0.05	0.04	0	0	0.04	0	0	0.81	0	0.05	0
Admiration	0	0.04	0.02	0.05	0	0	0	0	0	0.88	0	0.01
Contempt	0.01	0	0	0	0	0.03	0	0	0.05	0.05	0.86	0
Disgust	0.02	0.03	0	0	0.06	0.03	0.05	0.04	0.02	0	0	0.75

The score in the list is the highest.

We can see from the table that the flat-OCC+EMO-CBOW model we proposed can classify and identify the emotion of education public opinion data well. The proposed model accurately classified more than 65% of each emotion, with the “fault” category being the most accurate (88%). Overall, five out of 12 emotions were more than 80% accurate, accounting for nearly 50% of the total. There were ten numbers with an identification accuracy of more than 70%. Only two affective types were not well identified. These results prove that in the scholarly public opinion data set, the model we proposed can identify different emotional types well.

Tables 8, 9 show a confusion matrix for the 12-Class sentiment classification of the proposed model.

**Table 8:** 2-Class sentiment classification confusion matrix (Flat-OCC).

	**Negative**	**Positive**
Negative	0.85	0.15
Positive	0.14	0.86

**Table 9:** 2-Class sentiment classification confusion matrix (Flat-OCC+Emo-CBOW).

	**Negative**	**Positive**
Negative	0.88	0.12
Positive	0.13	0.87

We can see from the table that (1) the flat-OCC model we proposed can classify and identify the emotion of education public opinion data well; and (2) for the recognition of educational emotion classification, the categorization accuracy of the flat-OCC+EMO-CBOW model is significantly higher than that of a single flat-OCC model, which reveals that the emotional-pack-based model we propose can enhance our benchmark model.

## Conclusions

This paper studied the multiple problems of emotion detection based on the OCC emotion model in Chinese text, it analyzed and compared some standard methods and other attempts based on the model, it expounded on their advantages, disadvantages, and applications, and put forward its implementation scheme.

To apply the OCC model, we first tried to streamline appropriately. However, emotional expressions used widely in the text do not necessarily describe all elements. For example, “blessing” (best wishes) emotion may have happy associations for some, and the sentence does not necessarily contain specific events or references to indicate how it is being used, so strict accordance with the rules and variables may miss many possible situations. In Chinese text, especially in the microblog corpus full of the network language, the expression of the rules further expanded the richness of the corpus and enabled the experiment to be conducted. Improved models with 12 categories of emotions and rules are easier to use in formal linguistic representations.

Since this paper's work strongly depends on manual annotation corpus, subsequent attempts were made to build a corpus annotation tool that is easy to use and could reduce the workload of manual annotation. In order to verify the effectiveness and feasibility of the tool replacing manual work, a comparative experiment of labeling and forecasting was designed. The experimental data showed that this function could be used if there is a smaller workforce.

## Data availability statement

The raw data supporting the conclusions of this article will be made available by the authors, without undue reservation.

## Author contributions

The author confirms being the sole contributor of this work and has approved it for publication.

## Funding

This work was supported by the 2021 Major Bidding Fund of the 14th Five-Year Plan of Education and Science of Henan Province (2021JKZB15).

## Conflict of interest

The author declares that the research was conducted in the absence of any commercial or financial relationships that could be construed as a potential conflict of interest.

## Publisher's note

All claims expressed in this article are solely those of the authors and do not necessarily represent those of their affiliated organizations, or those of the publisher, the editors and the reviewers. Any product that may be evaluated in this article, or claim that may be made by its manufacturer, is not guaranteed or endorsed by the publisher.
